# A Trajectory of Thyroid Function: From Thyrotoxic Paralysis to Post-ablative Hypothyroidism

**DOI:** 10.7759/cureus.96924

**Published:** 2025-11-15

**Authors:** Janani Anantha Ganesan, Ngozi Maria Ekpemandu

**Affiliations:** 1 Acute Medicine, University Hospital Lewisham, Lewisham and Greenwich NHS Trust, London, GBR; 2 Internal Medicine, University Hospital Lewisham, Lewisham and Greenwich NHS Trust, London, GBR

**Keywords:** endocrine emergency, graves' disease, management of thyrotoxicosis, thyrotoxic hypokalemic periodic paralysis, thyrotoxicosis

## Abstract

Acute flaccid paralysis can obscure the underlying endocrine cause in thyrotoxic periodic paralysis (TPP), a rare but potentially life-threatening presentation of Graves’ disease. We describe a 23-year-old Cantonese man who presented with sudden bilateral proximal lower limb weakness and severe hypokalaemia after a high-carbohydrate meal. Subsequent investigations revealed thyrotoxicosis consistent with Graves’ disease. Management involved prompt potassium replacement, propranolol, and cautious initiation of carbimazole to avoid overtreatment. The patient later underwent radioactive iodine ablation, which successfully resolved thyrotoxicosis but led to post-ablative hypothyroidism requiring levothyroxine. This case underscores that TPP may represent the initial clinical expression of thyroid disease and highlights the critical importance of timely identification, meticulous management, and continued surveillance to mitigate iatrogenic risks.

## Introduction

Thyrotoxic periodic paralysis (TPP) is a rare endocrine emergency characterised by acute, reversible muscle weakness associated with hypokalaemia in the context of thyrotoxicosis [1,2]. Although it predominantly affects young Asian men, increasing global migration has led to a rise in cases among other ethnic groups, including Western populations [3-5]. The reported prevalence ranges from approximately 1%-2% among hyperthyroid patients of Asian descent to less than 0.1% in non-Asian cohorts [1,2,5,6].

The underlying pathology involves an intracellular shift of potassium caused by thyroid hormone-mediated upregulation of Na⁺/K⁺-ATPase activity, often precipitated by high-carbohydrate intake, strenuous exercise, or emotional stress [1,2,7-9]. Acute management focuses on cautious potassium replacement and the use of non-selective β-blockers to inhibit further intracellular potassium uptake, followed by definitive treatment of the underlying hyperthyroidism through antithyroid drugs, radioiodine therapy, or thyroidectomy [2,4,10,11].

Despite being a reversible condition, TPP is frequently misdiagnosed due to its rarity and overlap with neurological or primary electrolyte disorders [8-11]. Additional challenges include delayed recognition in non-endemic regions, risk of rebound hyperkalaemia from overcorrection, and potential hypothyroidism following definitive therapy [1,5,12]. We present a case of hypokalaemic TPP in a previously healthy individual, highlighting the importance of considering thyroid dysfunction in acute paralysis and ensuring careful endocrine monitoring during follow-up.

## Case presentation

A 23-year-old male of Cantonese heritage presented with sudden-onset bilateral lower limb weakness occurring shortly before 3 a.m. after consuming a late high-carbohydrate dinner. On trying to get up from his sofa, he noticed proximal weakness in both legs, making him unable to stand or walk without assistance. He denied any sensory disturbance, bowel or bladder incontinence or recent trauma. He had no significant past medical history, was not on any regular medications or supplements, and did not have any dietary restrictions or recent change in appetite. His father had a history of hypothyroidism, which required radioactive iodine therapy.

Upon arrival at the emergency department, the patient was alert and oriented. Vital signs were within normal limits except for mild tachycardia (heart rate 102 bpm). Neurological examination revealed symmetrical proximal muscle weakness in the lower limbs, with power graded as 3/5 on the Medical Research Council scale [13]. Distal lower limb muscles demonstrated slightly better strength, graded as 4/5. Deep tendon reflexes and sensation were preserved. Upper limb strength was normal. No cranial nerve deficits were observed. There was no palpable neck swelling or mass.

Investigations

Initial blood investigations revealed significant hypokalaemia (serum potassium: 2.1 mmol/L; reference range: 3.5-5.3 mmol/L) and mild metabolic acidosis (venous pH 7.33, bicarbonate 18 mmol/L). Thyroid function tests (Table 1) showed marked thyrotoxicosis with suppressed thyroid-stimulating hormone (TSH <0.01 mU/L; reference range: 0.4-4.0 mU/L), elevated free thyroxine (T4) level (serum free T4: 62.6 pmol/L; reference range: 9.0-25.0 pmol/L), and elevated free triiodothyronine (T3) level (serum free T3: 37.3 pmol/L; reference range: 3.5-7.8 pmol/L). Thyrotropin receptor antibodies (TRAb) were positive (TRAb: 10.33; reference range: <0.4 U/L: negative, 0.4-1 U/L: borderline positive, >1 U/L: positive), consistent with a diagnosis of Graves’ disease. Electrocardiography demonstrated a normal sinus rhythm.

**Table 1:** Biochemical Evolution of Thyroid Function in a Case of Thyrotoxic Periodic Paralysis Laboratory results demonstrating dynamic changes in thyroid function over time. Elevated serum free triiodothyronine (T3) and serum free thyroxine (T4) levels with suppressed thyroid-stimulating hormone (TSH) indicate hyperthyroidism, followed by a transition to hypothyroidism with markedly elevated TSH and low free T4. The presence of elevated thyroid receptor antibodies supports a diagnosis of Graves’ disease as the underlying aetiology.

Results	Reference range	07/11/23	08/11/23	30/04/24	18/06/24	10/09/24	26/11/24	29/05/25	18/06/25
Serum free T3	3.5-7.8 pmol/L	23.9		32.6					
Serum free T4	9.0-25.0 pmol/L	59.3		74.8	10.8	<3	18.4	<3.0	12.3
Thyroid-stimulating hormone	0.4-4.0 mU/L	<0.01		<0.01	<0.01	93.50	0.91	>100	84.90
Thyroid receptor antibody	<0.4 U/L: negative; 0.4-1.0 U/L: borderline positive; >1.0 U/L: positive		10.33						

**Figure 1:**
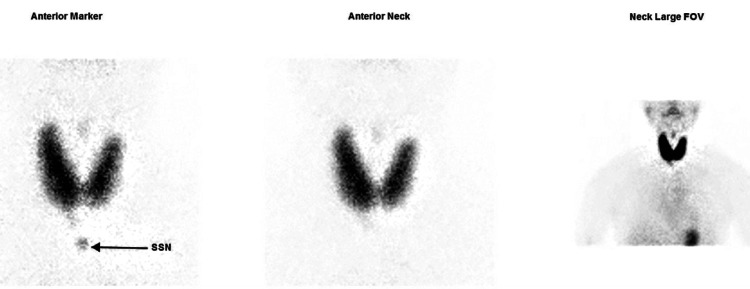
Thyroid Scintigraphy of the Patient With Thyrotoxic Periodic Paralysis Anterior planar scintigraphy images of the neck using radio-tracer Technetium-99m pertechnetate (Tc-99m) demonstrate increased and homogeneous radiotracer uptake in both thyroid glands which supports the diagnosis of Graves’ disease. Anterior Marker View: Localisation marker at the suprasternal notch (SSN) confirms anatomical orientation. Anterior Neck View: Focused view showing symmetric uptake in both thyroid lobes, consistent with diffuse toxic goitre of Graves’ disease. Neck Large Field-of-View (FOV): Broader anatomical context confirming absence of ectopic uptake or nodularity.

**Figure 2:**
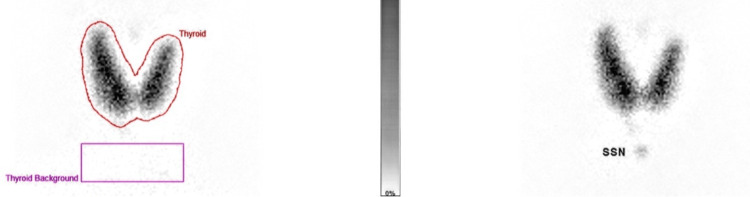
Thyroid Scintigraphy With Regions of Interest (ROI) Analysis Planar thyroid scintigraphy showing regions of interest (ROIs) for quantitative analysis. Left: ROI outlining the thyroid gland (red) and background region (purple) for uptake calculation. Right: Anatomical localization with suprasternal notch (SSN) marker. The grayscale bar indicates radiotracer uptake intensity, supporting assessment of thyroid function. Quantitative assessment revealed a thyroidal Technetium-99m pertechnetate (Tc-99m) uptake of 20.25% (reference range: 0.40%-4.00%), indicating globally increased thyroid function.

A technetium-99m (Tc-99m) pertechnetate thyroid scan showed diffusely increased tracer uptake throughout both thyroid lobes (Figure 1). Quantitative assessment revealed a thyroidal uptake of 20.25% (reference range: 0.40%-4.00%), indicating globally increased thyroid function (Figure 2). No focal hot or cold nodules were observed.

**Figure 3:**
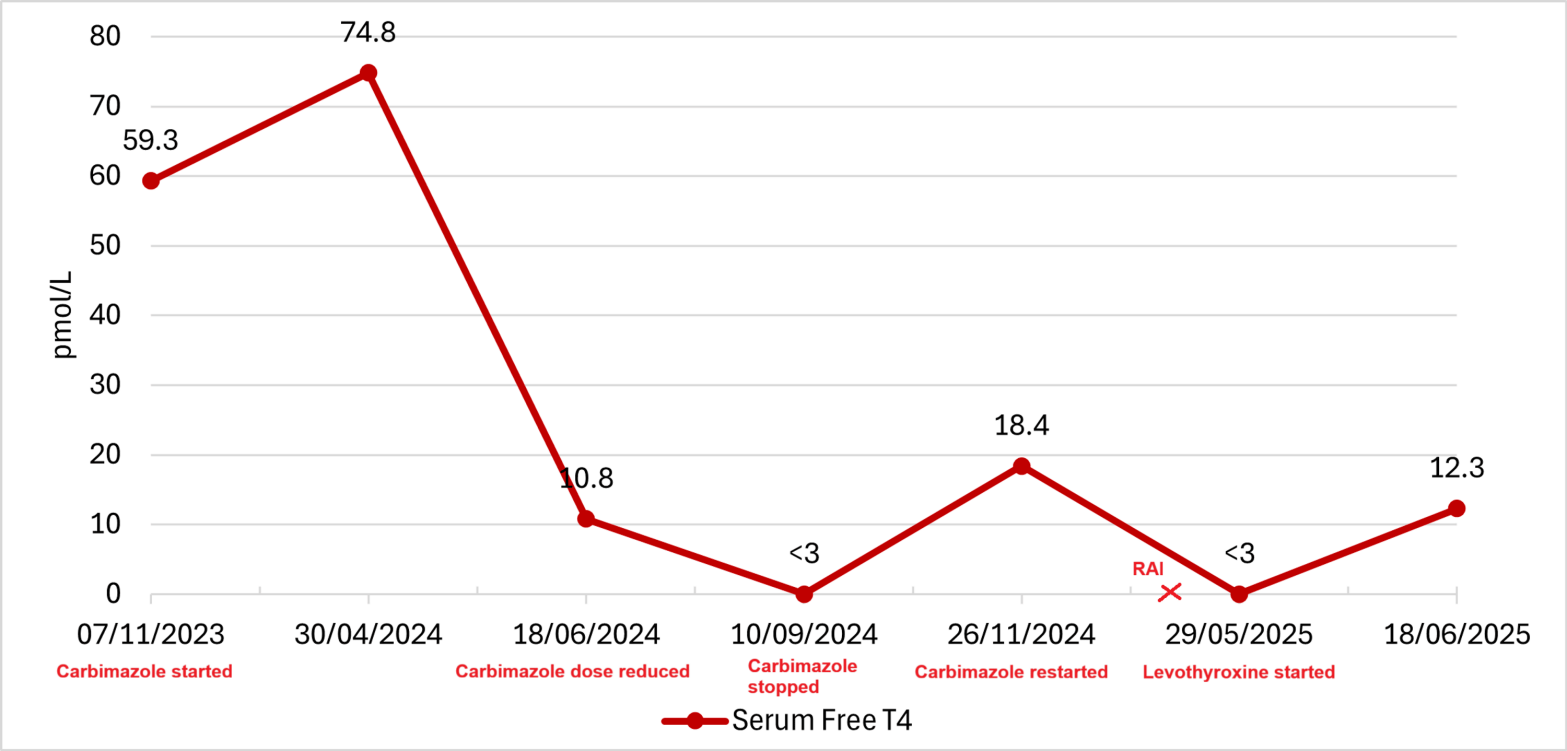
Serum Free T4 Trajectory in Relation to Key Therapeutic Interventions Line graph showing serum free T4 concentrations (in pmol/L) from November 2023 to June 2025. Initial thyrotoxicosis (59.3-74.8 pmol/L) improved following carbimazole initiation in November 2023. Carbimazole dose reduction in June 2024 was prompted by normalisation of free T4 levels (10.8 pmol/L), and treatment was discontinued in September 2024 due to iatrogenic hypothyroidism (<3 pmol/L). To prevent recurrence of thyrotoxicosis, carbimazole was restarted in November 2024. Radioactive iodine (RAI) therapy administered in March 2025 induced hypothyroidism, as evidenced by markedly reduced free T4 levels in May 2025 (<3 pmol/L). Thyroid function subsequently improved following levothyroxine supplementation. Free T4 reference range: 9.0-25.0 pmol/L; treatment milestones are annotated.

**Figure 4:**
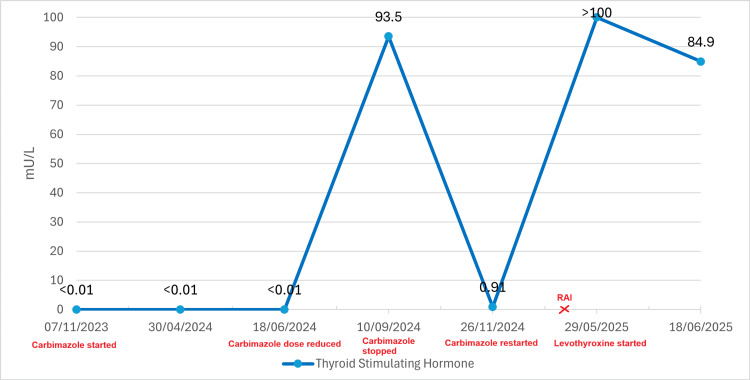
Serum Thyroid-Stimulating Hormone (TSH) Trajectory in Relation to Key Therapeutic Interventions Line graph showing serum TSH concentrations (mU/L) from November 2023 to June 2025 in a patient with thyrotoxic periodic paralysis. TSH was suppressed (<0.01 mU/L) due to hyperthyroidism before carbimazole initiation (November 2023). Carbimazole dose was reduced in June 2024 despite persistent TSH suppression (<0.01 mU/L). A sharp rise to 93.5 mU/L was seen in September 2024, indicating iatrogenic hypothyroidism, which prompted treatment cessation. TSH transiently normalised (0.91 mU/L), and carbimazole was restarted in November 2024 to prevent recurrence of thyrotoxicosis. Following radioactive iodine therapy (March 2025), TSH rose again (>100 mU/L) preceding levothyroxine replacement (May 2025). TSH reference range 0.4-4.0 mU/L; treatment milestones are annotated.

Serial monitoring of thyroid hormone levels was performed throughout treatment (Table 1), including the period following radioactive iodine therapy. The patient's biochemical response is illustrated in a trend graph showing free T4 and TSH levels (Figures 3, 4).

Treatment

Management included intravenous potassium chloride infusion to correct hypokalaemia, oral propranolol (40 mg) to counteract adrenergic overactivity and inhibit Na⁺/K⁺-ATPase activity, and initiation of carbimazole (20 mg twice daily) to reduce thyroid hormone synthesis. The patient’s muscle strength improved markedly following potassium correction, and symptoms resolved within 24 hours.

He was subsequently reviewed in the endocrine clinic, where antithyroid therapy was titrated based on serial thyroid function tests. After a multidisciplinary team meeting and patient discussion, he underwent definitive treatment with a single oral dose of radioactive iodine (RAI) therapy (654-MBq) without acute complications. Follow-up blood tests revealed hypothyroidism, for which levothyroxine replacement therapy was initiated and adjusted to maintain euthyroid status. He continues to be monitored regularly with thyroid function tests and has not experienced recurrence of paralytic symptoms.

## Discussion

Thyrotoxic periodic paralysis (TPP) is a rare but well-documented complication of thyrotoxicosis, primarily associated with Graves' disease. Like previous reports by Kung [1] and by Pothiwala and Levine [2], it typically presents with acute-onset painless muscle weakness and biochemical hypokalaemia. Although hyperthyroidism is more common in women, TPP disproportionately affects young males, particularly those of Asian descent [1,2]. The current case aligns with this demographic trend and highlights the classical presentation of TPP following a high-carbohydrate meal in a young Cantonese male.

The pathophysiology of thyrotoxic periodic paralysis (TPP) involves a rapid intracellular shift of potassium due to overstimulation of the Na⁺/K⁺-ATPase pump. Thyroid hormone enhances both the expression and activity of this pump, and its effect is synergistically amplified by insulin and catecholamines. Heavy exercise elevates catecholamines [3], while high-carbohydrate meals induce insulin surges [4], together accelerating potassium influx into muscle cells. This cascade results in acute hypokalaemia and flaccid paralysis. In this case, the patient experienced symptoms shortly after consuming a carbohydrate-rich dinner, supporting existing evidence that postprandial insulin surges may trigger attacks [2,5].

Clinically, TPP may mimic other causes of acute flaccid paralysis, such as Guillain-Barré syndrome or familial periodic paralysis. However, the absence of sensory loss or cranial nerve involvement and biochemical evidence of hypokalaemia and thyrotoxicosis are key differentiators [4]. A key differential diagnosis is familial hypokalaemic periodic paralysis, which typically lacks features of thyrotoxicosis. In our case, the presence of clinical and biochemical hyperthyroidism and positive thyroid receptor antibodies (TRAb) confirmed Graves’ disease, effectively ruling out hypokalaemic periodic paralysis. Our patient’s presentation was consistent with the classical profile of TPP. Similar diagnostic challenges have been reported by Gubran et al. [3], where a young man developed acute paralysis following vigorous exercise, and by Askar et al. [6], who described TPP as a late complication of Graves’ disease following COVID-19 infection.

Prompt management involves cautious correction of hypokalaemia, typically with intravenous potassium, alongside beta-blockade using non-selective agents such as propranolol. This approach mitigates the activity of the Na⁺/K⁺-ATPase and helps reverse paralysis. Our patient responded well to this regimen. Subsequent initiation of antithyroid therapy with carbimazole and definitive radioactive iodine (RAI) treatment mirrored management protocols reported in the literature [1,4,7].

Importantly, long-term follow-up is necessary to monitor for post-treatment hypothyroidism, as seen in our patient. Levothyroxine replacement should be initiated based on regular thyroid function testing to maintain a euthyroid state and prevent further neuromuscular complications [1,5,12]. As highlighted by Kung [1], achieving and maintaining euthyroidism is key to preventing recurrence.

In summary, this case reinforces the importance of considering TPP in patients presenting with acute hypokalaemic paralysis, particularly in young men of Asian background. Early recognition, identification of triggers such as carbohydrate intake, and appropriate endocrine management are essential for optimal outcomes. Timely diagnosis not only prevents recurrent attacks but also reduces the risk of serious complications such as cardiac arrhythmias and respiratory compromise.

## Conclusions

Thyrotoxic periodic paralysis (TPP) represents a rare but clinically significant complication of thyrotoxicosis, often presenting with acute hypokalaemic paralysis in the absence of overt hyperthyroid symptoms. This case underscores the necessity of maintaining a high index of suspicion for endocrine aetiologies in patients with sudden-onset muscle weakness. Thyroid function testing should be routinely included in the initial evaluation of acute paralysis to avoid diagnostic delays. Management requires prompt yet judicious potassium replacement and non-selective beta-blockade to mitigate Na⁺/K⁺-ATPase activity. While definitive treatment of Graves’ disease resolves the underlying thyrotoxicosis, it obligates lifelong surveillance for iatrogenic hypothyroidism. Early recognition and targeted endocrine therapy are essential to prevent recurrence and reduce the risk of long-term neuromuscular and cardiovascular complications.
